# Uncovering the mechanism of banana “green ripening”

**DOI:** 10.1093/plcell/koad048

**Published:** 2023-02-16

**Authors:** Peng Liu

**Affiliations:** Assistant Features Editor, The Plant Cell, American Society of Plant Biologists, USA; Donald Danforth Plant Science Center, Saint Louis, MO 63146, USA

**Figure 1.**
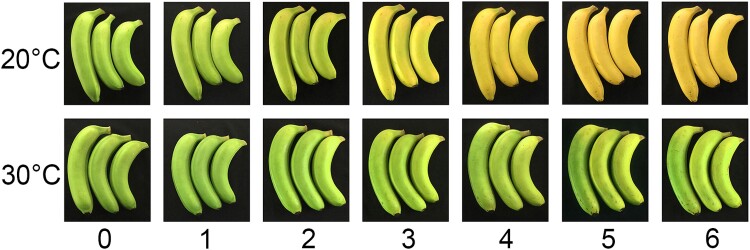
Green ripening of banana under high temperature. Appearance of banana peel during ripening at 20 and 30 °C, from 0 to 6 d after ethylene treatment. Bananas ripened at 30 °C remained green, indicating a higher retention of chlorophyll in the peel at high temperature. Adapted from Wei et al. (2023), Fig. 1.

The green-to-yellow color of banana peels is an excellent indicator of fruit ripening. We can easily select ready-to-eat bananas from the grocery store just by looking at them. However, the color can trick us sometimes if the bananas are not stored properly. A banana with green peels may become soft and overripe at high temperatures (Seymour et al. 1987). This phenomenon is called “green ripening” (see Fig. 1). Research conducted by **Wei Wei and colleagues (Wei et al. 2023)** unveiled a new molecular mechanism underlying this coloring process inside banana cells.

Their study started with examining the expression of 9 chlorophyll catabolic genes (CCGs) during banana ripening. The color of banana peels is associated with chlorophyll content, which degrades during the ripening process (Drury et al. 1999). The authors found the expression of 5 CCGs greatly reduced at 30 °C compared with 20 °C. All 5 genes can promote chlorophyll degradation, and one of them, *MaSGR2*, has a dominant effect. Using a yeast one-hybrid screening assay, the authors identified a transcription factor MaMYB60 that directly bound to the promoter of *MaSGR2*.

Further analysis showed that MaMYB60 was a positive regulator of chlorophyll degradation through activating all 5 CCGs. A transient transformation assay of MaMYB60 functions in banana peels showed a correlation between the color index and MaMYB60 expression levels. A negative correlation was also observed between chlorophyll content and MaMYB60 expression. The next question addressed was whether the expression of MaMYB60 is under the control of high temperature. Significantly lower levels of both the mRNA and protein of MaMYB60 were observed at 30 °C than at 20 °C. The authors were particularly interested in MaMYB60 protein stability since this protein level was highly correlated with and CCG transcript levels, chlorophyll content, and color index.

Based on the above results, the authors hypothesized that ubiquitin-mediated proteolysis is involved in the enhanced degradation of MaMYB60 under high temperatures. With considerable effort, they identified a RING-type ubiquitin E3 ligase MaBAH1 that mediated the degradation of MaMYB60 directly. The specificity of ubiquitin-mediated proteolysis is usually defined by the E3 ligase, which recognizes and interacts with the target protein (Scheffner et al. 1995). Here, the authors used multiple techniques to verify that MaBAH1 ubiquitinated MaMYB60, targeting it for temperature-dependent proteasomal degradation.

Their proposed model starts with the induction of MaBAH1 by high temperatures. As a ubiquitin E3 ligase, MaBAH1 recognizes MaMYB60 proteins and accelerates its degradation. MaMYB60 is a positive regulator of key chlorophyll CCGs, and less MaMYB60 under high temperatures affects chlorophyll metabolism, leading to a slower disappearance of green color in banana peels. This ubiquitin-mediated regulatory pathway explains why bananas do not turn yellow under high temperatures. Future studies may seek to uncover the complete signal transduction pathway from high temperatures to MaBAH1 expression. The authors also identified multiple banana genes involved in fruit degreening. In this era of genome editing, these genes could be tested and utilized as potential breeding targets for improving banana storage and fruit quality.
